# Bandgap Engineering of 2D Materials toward High-Performing Straintronics

**DOI:** 10.1021/acs.nanolett.4c03321

**Published:** 2024-10-02

**Authors:** Conor S. Boland, Yiwei Sun, Dimitrios G. Papageorgiou

**Affiliations:** † School of Mathematical and Physical Sciences, University of Sussex, Brighton, BN1 9QH, U.K.; ‡ School of Engineering and Materials Science, Queen Mary University, London, E1 4NS, U.K.

**Keywords:** Straintronics, Bandgap Engineering, 2D Materials, Mechanical Strain, Transition Metal Dichalcogenides (TMDs), Flexible Electronics

## Abstract

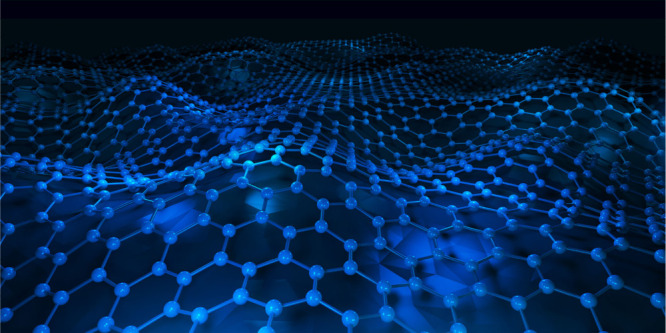

Straintronics leverages mechanical strain to alter the electronic properties of materials, providing an energy-efficient alternative to traditional electronic controls while enhancing device performance. Key to the application of straintronics is bandgap engineering, which enables tuning of the energy difference between the valence and conduction bands of a material to optimize its optoelectronic properties. This mini-review highlights the fundamental principles of straintronics and the critical role of bandgap engineering within this context. It discusses the unique characteristics of various two-dimensional (2D) materials, such as graphene, transition metal dichalcogenides (TMDs), hexagonal boron nitride (h-BN), and black phosphorus, which make them suitable for strain-engineered applications. Detailed examples of how mechanical deformation can modulate the bandgap to achieve desired electronic properties are provided, while recent experimental and theoretical studies demonstrating the mechanisms by which strain influences the bandgap in these materials are reviewed, emphasizing their implications for device fabrication. The review concludes with an assessment of the challenges and future directions in the development of high-performing straintronic devices, highlighting their potential applications in flexible electronics, sensors, and optoelectronics.

Straintronics, a subfield of nanoelectronics, harnesses mechanical strain to modify the electronic properties of materials. This approach provides a pathway to enhance device performance with reduced energy consumption and simplified fabrication processes. The fundamental principle of straintronics involves the application of mechanical deformation to a material, thus altering its electronic band structure and consequently, its electrical, optical, and magnetic properties. Straintronics have shown considerable promise in applications such as flexible electronics, sensors, and memory devices.^1,2^ Bandgap engineering is key for the successful implementation of straintronic devices. The bandgap of a material, defined as the energy difference between its valence band and conduction band, is crucial in determining its electrical conductivity and optical absorption properties. By finely tuning the bandgap, materials can be optimized for specific applications, resulting in enhanced efficiency and performance.

Two-dimensional materials have revolutionized material science due to their unique structural, electronic, and mechanical properties. In the context of 2D materials, bandgap engineering through the application of strain provides an effective method to achieve desired electronic properties without the need for chemical doping or complex fabrication techniques. Graphene may be the most well-known 2D material that exhibits extraordinary electrical conductivity^3^ and mechanical properties^4^ (among others); however, its zero bandgap limits its application in certain electronic devices (e.g., transistors and LEDs). This limitation has driven the exploration of a multitude of 2D materials such as TMDs with a chemical composition of MX_2_ where M is a transition metal and X is S, Se and Te (also displaying different structural phases e.g. 1T, 1 Td, 1T′ and 2H), h-BN, and black phosphorus, which possess intrinsic bandgaps suitable for various electronic and optoelectronic applications. In straintronics, 2D materials are particularly advantageous due to their high flexibility and ability to sustain significant mechanical strain without failure. For instance, monolayer MoS_2_, undergoes a direct-to-indirect bandgap transition when subjected to ∼1% tensile strain, significantly altering its electronic properties.^5^ Similarly, strain can modulate the bandgap of black phosphorus, making it a versatile material for strain-engineered devices.^6^ The atomically thin nature and flexibility of these materials allows to precisely control strain, enabling fine-tuning of their electronic properties through mechanical deformation.

The bandgap in 2D materials is a critical factor that dictates their suitability for electronic applications. Unlike bulk materials, 2D materials exhibit bandgaps that are highly sensitive to external factors such as strain, electric field and chemical modifications. These degrees of freedom allow for dynamic tuning of their electronic properties, which is essential for the development of high-performance, strain-engineered devices. For example, although graphene is gapless, various methods have been developed to induce a bandgap such as chemical functionalization (hydrogenation), introduction of defects, substrate engineering, nanopore formation and formation of graphene-based van der Waals heterostructures.^7−9^ Also, patterning graphene into its allotropes, such as nanoribbons, can open a bandgap due to quantum confinement effects.^10,11^ TMDs like MoS_2_, WS_2_, and WSe_2_ are known for their tunable bandgaps which can be function of layer number, strain, chemical composition and their heterostructures. Applying uniaxial strain to monolayer WS_2_ can shift its bandgap from direct to indirect at ∼2.5% strain, significantly affecting its photoluminescence properties.^12^ This tunability is exploited in flexible and wearable electronics. Black phosphorus (BP) exhibits a layer-dependent bandgap, which can be further tuned by applying strain. BP bandgap ranges from 0.3 eV (bulk) to around 2.0 eV (monolayer)^13,14^ as a result of the differences in electronic state coupling among BP layers; however, the application of strain in the basal plane of BP mostly affects in-plane bonding and therefore there is little dependence on layer thickness. Zhang et al.^15^ have reported a 20% modulation of the bandgap of 6-layer BP with only 1% uniaxial strain, making it a promising candidate for strain-sensitive photodetectors and transistors. In addition, while h-BN is typically used as an insulating layer due to its wide bandgap, introducing strain can slightly modify its electronic properties, enhancing its compatibility with other 2D materials in heterostructures.^16^ Nevertheless, the discovery and synthesis of novel 2D materials continue to expand the possibilities for straintronic applications. A prime example is the recent experimental realization of semiconducting monolayer Si_2_Te_2_ films, which were successfully grown on Sb_2_Te_3_ thin-film substrates.^17^ This achievement is particularly noteworthy because Si_2_Te_2_ does not exist naturally in bulk form, and its monolayer structure was stabilized through substrate-induced strain. The strain not only stabilized the material but also endowed it with a mid-infrared bandgap, making it highly suitable for optoelectronic applications in this spectral range. The ability to engineer such a bandgap through careful control of the substrate-induced strain highlights the potential of strain engineering in creating new 2D materials with tailored properties that do not have bulk counterparts.

This mini-review aims to provide an overview of the current advancements in bandgap engineering of 2D materials toward high-performing straintronics. The primary objectives are to present the fundamental principles of straintronics and the critical role of bandgap engineering in this context, examine the unique properties of various 2D materials that make them suitable for strain-engineered applications, and provide detailed examples of bandgap modulation through strain. Additionally, we aim to review recent experimental studies that highlight the mechanisms by which strain influences the bandgap in 2D materials and the practical implications of these findings in device fabrication. Finally, we seek to identify key applications of strain-engineered 2D materials in flexible electronics, sensors, and optoelectronics, and discuss the challenges and future directions in the field.

## Fundamentals of Bandgap Engineering

### Bandgap Basics

**Figure 1:**
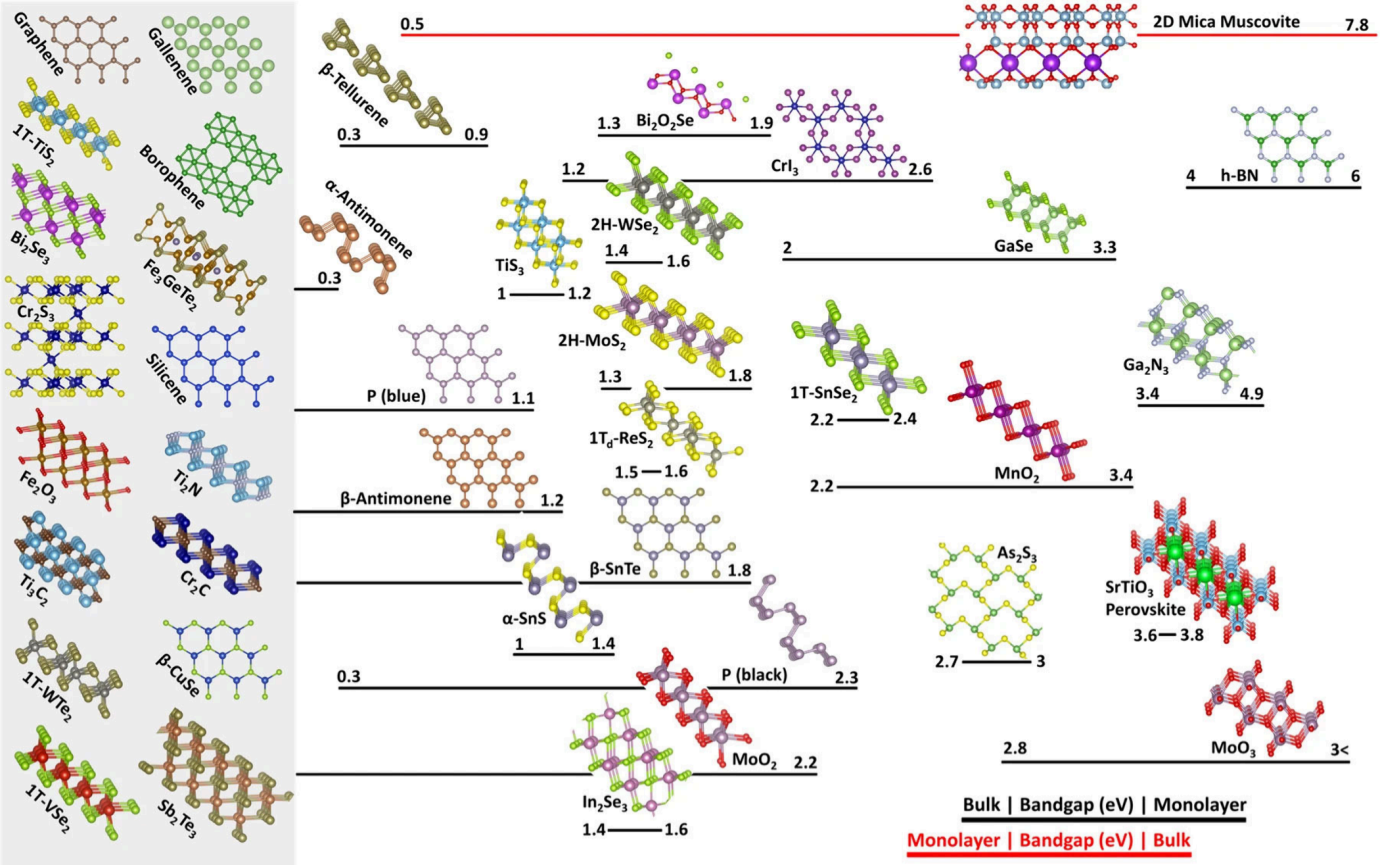
A number of 2D crystal structures including their bandgaps, guided by their bandgap range and scale (from bulk to monolayer). The 2D materials listed in the gray box on the left are either zero or near-zero bandgap, metallic or semimetallic. Adapted with permission from ref (18). Copyright 2020, Springer Nature Limited.

The concept of a bandgap is fundamental in understanding the electronic properties of 2D materials. The bandgap of a material is the energy difference between the valence band (VB), which is the highest energy range fully occupied by electrons, and the conduction band (CB), which is the lowest energy range that is partially occupied. This gap determines how a material will interact with electrical and optical stimuli. For instance, a large bandgap results in an insulator, where electron movement across the bandgap is restricted, and only significant energy input can induce conductivity. Conversely, a small or non-existent bandgap, as seen in conductors like graphene, facilitates free electron flow even at low energy levels, promoting high electrical conductivity. In semiconductors such as TMDs, electrons are excited from the VB to the CB when they absorb energy that meets or exceeds the electronic band gap. This process leaves behind unoccupied states (holes) in the VB; as a result, the bandgap is moderate, allowing for controlled electron movement, essential for applications such as transistors and photonic devices. Different 2D materials exhibit different bandgap characteristics as a function of their structure, layer number, strain, functional groups and edge properties. For example, TMDs typically have direct bandgaps that are suitable for optoelectronic devices, whereas black phosphorus can have a highly tunable bandgap, making it versatile for various electronic applications. Low and co-workers, in their excellent review^18^ have nicely summarized the bandgaps of multiple 2D materials, presented in Figure 1, demonstrating clearly the range of bandgaps of 2D materials as a function of layer number.

### Mechanisms of Bandgap Tuning

Bandgap tuning refers to the modification of the bandgap to achieve desired electronic properties. This can be accomplished through various mechanisms, including chemical doping, quantum confinement, and strain engineering. Chemical doping involves introducing impurities into a material to alter its electronic structure. Quantum confinement occurs in nanostructures, where reducing the size of the material to the nanoscale leads to discrete energy level formation and an increased bandgap due to the spatial confinement of charge carriers (electrons and holes). Strain engineering is a particularly effective method for bandgap tuning, especially in 2D materials, since the bond lengths and angles between atoms are changed under strain. This alteration affects the overlap of atomic orbitals, which in turn changes the electronic band structure. The mechanism of strain effects on the bandgap can be understood from three aspects:

#### Bonding and Anti-Bonding States as the Underlying Physics

Tensile strain typically reduces the band gap by increasing the atomic spacing, which lowers the energy difference between the conduction and valence bands. The underlying physics involves the concept of bonding and antibonding states. Bonding states are formed when atomic orbitals overlap constructively, leading to lower energy states that make up the valence band, while anti-bonding states result from destructive overlap, creating higher energy states that constitute the conduction band.

As strain increases the distance between atoms, it reduces orbital overlap. This decreases the stabilization of bonding states (raising their energy) and decreases the destabilization of antibonding states (lowering their energy), which narrows the energy gap between them, thus reducing the band gap.

#### Exceptions and Complexities in Real Materials

While bonding and antibonding states provide a useful framework for understanding strain effects, the actual behavior in many materials, especially complex ones like graphene, is more nuanced.^19^ The states near the band edges are not always purely bonding or antibonding. The band structure is influenced by multiple factors, including crystal symmetry, electron–electron interactions, and the specific nature of the atomic orbitals involved. In graphene, for instance, the π (bonding) and π* (antibonding) bands are derived from p-orbitals, but the states near the Dirac points (the band edges) result from a unique linear dispersion relationship rather than simple bonding or antibonding interactions.^19^

#### Limitations of the Bonding/Anti-Bonding Concept – The Case of Graphene

Graphene provides a clear example of where the bonding and antibonding framework falls short. In graphene, the π and π* states are part of the same continuous band structure, and they are not spatially separable in the way they might be in simpler systems. The distinction between these states is less clear-cut because they are interconnected through the crystal’s symmetry and topology. The conduction and valence bands touch at the Dirac points, a feature arising from graphene’s unique band structure rather than simple bonding/antibonding distinctions.^20^ In addition, while bonding and antibonding states share the same quantum numbers (e.g., angular momentum, spin) when they originate from the same atomic orbitals, in simple molecular systems, their distinction lies in their energy levels and the spatial distribution of electron density due to constructive versus destructive interference of wave functions. In solids like semiconductors, the states at the edges of the conduction and valence bands often do not have the same quantum numbers because they are formed from different sets of atomic orbitals or due to band structure complexities. However, in graphene, the π and π* bands form a continuous spectrum, making the distinction between bonding and antibonding states less applicable. This continuous band structure, where conduction and valence bands touch at the Dirac points, is more a result of graphene’s symmetry and topology rather than traditional bonding or antibonding interactions.^21^ To further demonstrate the strain effects on the band gap in 2D materials, we examine graphene as a case study. Figure S1 (Supporting Information) illustrates the total valence charge density of bilayer graphene, along the (110) plane for Bernal stacking.^22^ The delocalized nature of the π-orbitals is clearly visible, and it is evident that the π and π* states are not spatially separable as they might be in simpler molecular systems. This lack of spatial separation complicates the application of the bonding and antibonding framework typically used to understand band structure modifications under strain.

To quantitatively assess the impact of strain, Figure S2 presents the density of states (DoS) for graphene under varying strain conditions and different substrate interactions.^23^ The graphene was strained to match the lattice of the silicon substrates, with isotropic tensile strain applied on Si (111) and anisotropic strain on other surfaces. For instance, graphene on Si (110) experiences both compressive and tensile strains. The figure demonstrates that for free-standing graphene, band gap opening is not primarily due to strain but rather due to the breaking of symmetry through anisotropic strain, which is further significantly modified by substrate interactions. These findings highlight the complexity of predicting strain effects on the band gap in real-world scenarios where substrate interactions are inevitable.

In 2D materials, strain can be applied through various means, such as bending, stretching, or compressing the material. The effect of strain on the bandgap depends on the type of strain (uniaxial or biaxial), its magnitude, and direction. For example, in monolayer MoS_2_, applying uniaxial tensile strain along the armchair direction can reduce the bandgap, while compressive strain can increase it. However, high levels of compressive strain can lead to early buckling, limiting the tunability of the band gap. This tunability arises because strain alters the orbital overlap between atoms, which in turn affects the energy levels of the valence and conduction bands.

Graphene is inherently a gapless material, which limits its use in various technologies unless an energy gap is created at the K and K’ points in the Brillouin zone. Early first-principles calculations indicated that a bandgap in graphene could be achieved with just 1% uniaxial strain.^24^ However, this finding was later revised, as it was found that the Dirac cone slightly shifts from the special K or K’ points in the reciprocal space. Through tight-binding approaches and density functional theory (DFT) calculations, it has been realized that a strain higher than 20% needs to be implemented along the zigzag direction to open a spectral gap. These high levels of strain can be impractical for implementation in many cases.^25^

In summary, while strain engineering is a powerful tool for bandgap tuning, the actual effect on the band structure can be complex and material-dependent, often requiring detailed computational and experimental analysis to fully understand and utilize. 2D materials, such as graphene, with their unique electronic properties, exemplify the challenges and nuances in applying strain to induce a bandgap.

### Strain Engineering and Valleytronics in 2D Materials

Valleytronics, a field that exploits the valley degree of freedom in 2D materials, has gained significant attention due to its potential to complement traditional electronics and spintronics.^26−28^ In materials like TMDs such as MoS_2_, WS_2_, and WSe_2_, electrons can occupy distinct energy valleys in momentum space, typically at the K and K’ points in the Brillouin zone. These valleys can be selectively populated using circularly polarized light, and their dynamics can be controlled by various external factors, including strain.

Strain engineering, when combined with valleytronics, opens up new avenues for manipulating the electronic and optical properties of 2D materials. By applying strain, it is possible to modify the energy difference between valleys, thereby altering the valley polarization and valley coherence properties. For instance, strain can induce shifts in the energy levels of the K and K’ valleys, leading to changes in the exciton energies and enabling control over the polarization of emitted light. This tunability is critical for developing devices that rely on valley-selective operations, such as valley transistors and valley-based quantum computing elements.^26^

Moreover, strain can modulate intervalley scattering rates, which are crucial for maintaining valley coherence over longer distances—a key requirement for valleytronic devices. By carefully engineering the strain, it is possible to suppress intervalley scattering, thereby enhancing valley coherence and improving device performance. Additionally, the coupling between strain and valley degrees of freedom can lead to novel phenomena, such as valley-dependent piezoelectric effects, where the piezoelectric response varies depending on the valley polarization.

The integration of valleytronics and straintronics in 2D materials represents a powerful strategy for designing next-generation electronic and optoelectronic devices. This approach leverages the properties of 2D materials, where both the electronic band structure and valley dynamics can be finely tuned by mechanical deformation, offering unprecedented control over device functionalities. Future research in this area will likely focus on optimizing the strain-valley interaction to achieve even greater control over electronic and optical properties, paving the way for innovative applications in quantum computing, information processing, and beyond.

## 2D Materials for Straintronic Bandgap Engineering

### Sample Preparation

**Figure 2:**
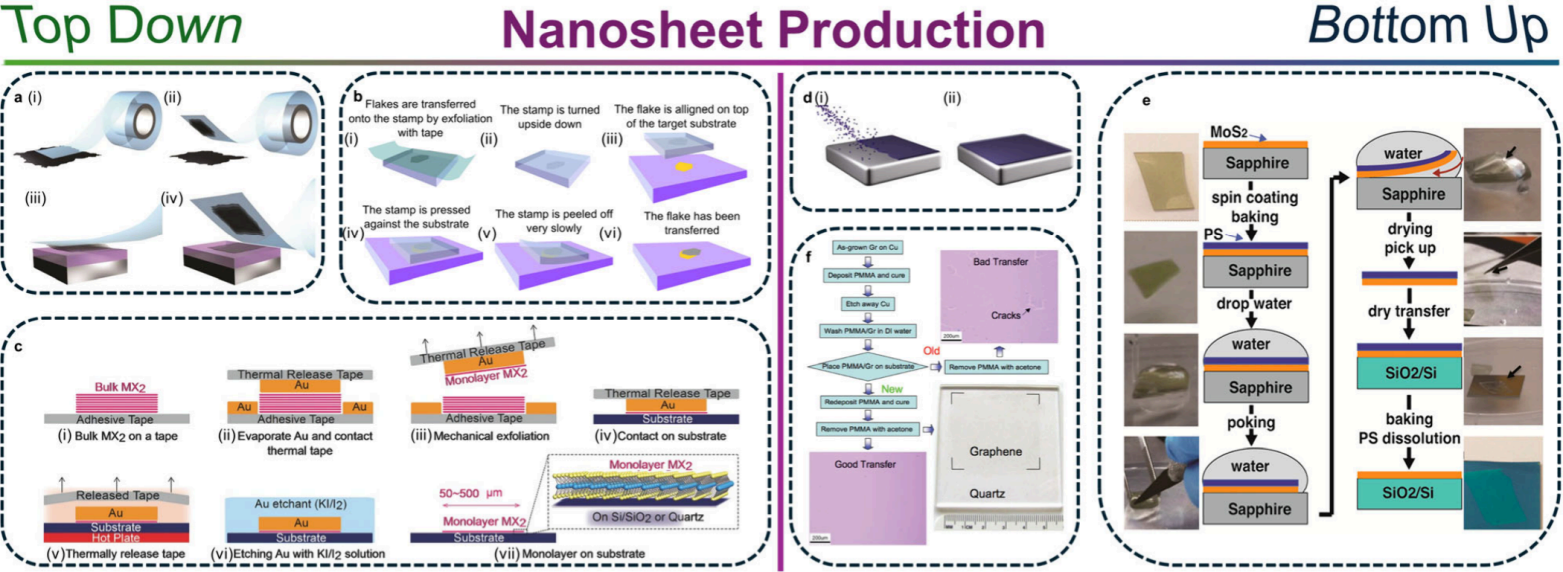
Production methods for nanosheets. (**a**) Micromechanical exfoliation of 2D crystals. (i) Adhesive tape is pressed against a 2D crystal so that the top few layers are attached to the tape (ii). (iii) The tape with crystals of layered material is pressed against a surface of choice. (iv) Upon peeling off, the bottom layer is left on the substrate. Reproduced with permission from,^94^ Copyright 2012, IOP Publishing. (**b**) Diagram of the steps involved in the preparation of the viscoelastic stamp and the deterministic transfer of an atomically thin flake onto a user-defined location (for instance another atomically thin flake). Reproduced with permission from ref (95). Copyright 2014, IOP Publishing. (**c**) Schematic illustration of the Au exfoliation process. Adapted with permission from ref (96). Copyright 2016, WILEY-VCH Verlag GmbH & Co. KGaA, Weinheim. (**d**) 2D crystals are grown by CVD on a surface of a metal (i) and (ii). Reproduced with permission from ref (94). Copyright 2012, IOP Publishing. (**e**) Illustration of the surface-energy-assisted transfer process. Typical images of the transfer process. The arrows point toward the MoS_2_ film for visual convenience. Adapted with permission from ref (36). Copyright 2014 American Chemical Society. (**f**) Processes for transfer of graphene films (“Gr” = graphene). The top-right and bottom-left insets are the optical micrographs of graphene transferred on SiO_2_/Si wafers (285 nm thick SiO_2_ layer) with “bad” and “good” transfer, respectively. The bottom-right is a photograph of a 4.5 × 4.5 cm^2^ graphene on quartz substrate. Reproduced with permission from ref (37). Copyright 2009 American Chemical Society.

Owing to weak van der Waals (vdW) forces binding layers together in bulk layered crystals, single layers can be easily isolated through *top-down* techniques (Figure 2a-c). Most commonly for straintronic research, mechanical exfoliation is utilized to yield few to single layered, large aspect ratio nanosheets.^29,30^ This process is simple in nature and requires the application of an adhesive tape,^31^ a soft flexible polymeric layer,^32^ or gold-mediated interface^33^ to the surface of a bulk crystal segment. Through repeated adhesion/peeling cycles, material is then gradually exfoliated, with nanosheets eventually isolated. Using stamping, nanosheets can then be transferred onto the desired substrate.^34^ Alternatively, monolayers of semiconducting nanosheets can also be grown through *bottom-up* procedures (Figure 2d), most commonly chemical vapor deposition (CVD).^35^ Through sacrificial growth substrates and polymer coatings, grown nanosheets can be transferred via surface-energy-assisted processes^36^ or wet etching^37^ to a secondary one (Figure 2e and f respectively). Though both material production methods are highly versatile, intrinsically, the procedures do result in the ubiquitous presence of polymeric films coating the nanosheets which may affect properties.^38^ Additionally, environmental conditions can also result in the degradation of nanosheets^39^ and an unintentional shifting in lattice constant^40^ due to oxidation.

### Methods for Applying Strain to Nanosheets

**Figure 3:**
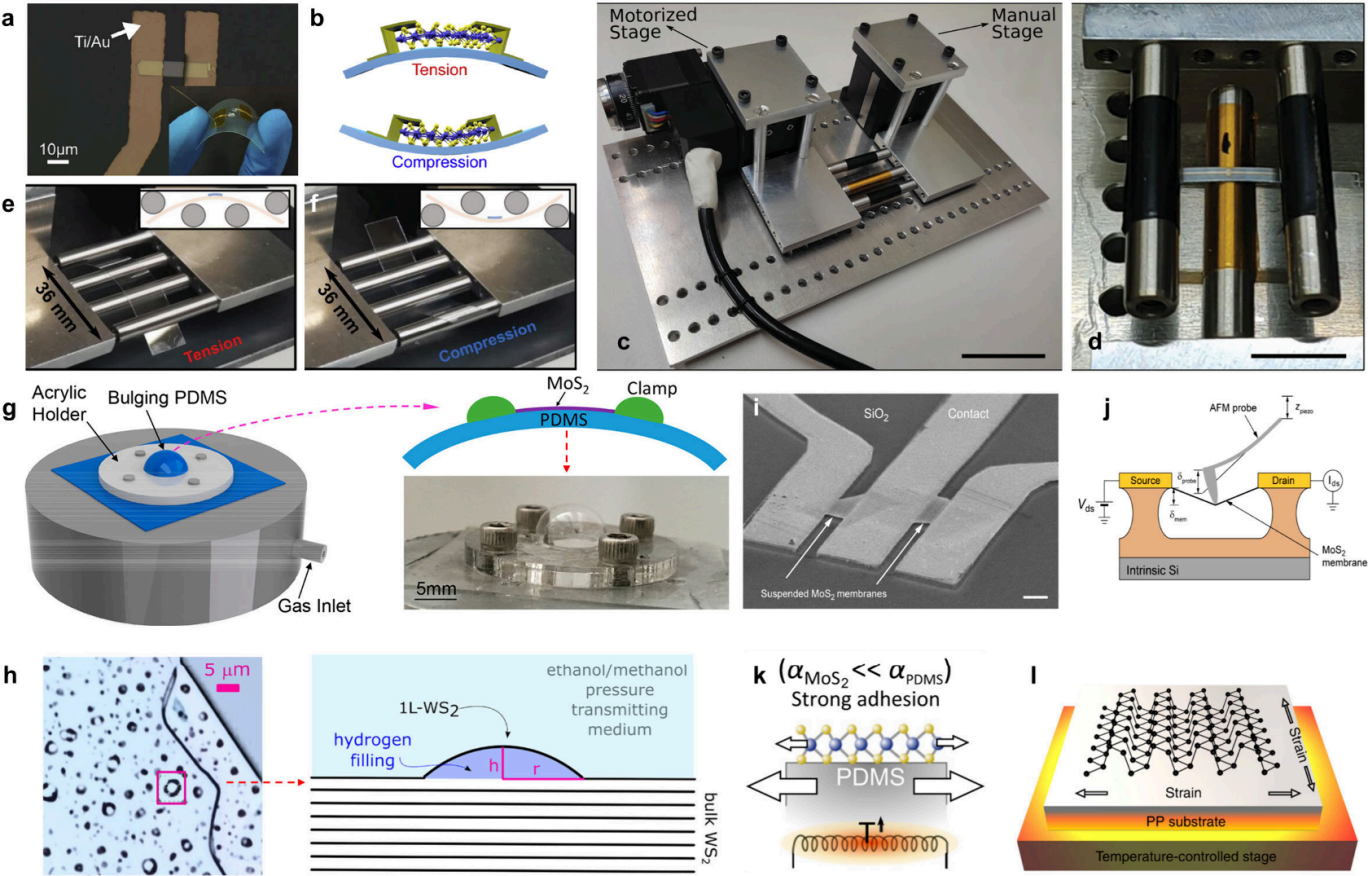
Nanosheet deformation procedures. (**a**) Optical image of a ReSe_2_ device on the flexible polyethylene naphthalate substrate. (**b**) Schematic illustration of the variable tensile/compressive strains induced by upward/downward bending of the flexible substrate via two-point flexation. Adapted with permission from ref (61). Copyright 2020 Elsevier B.V. (**c**) Picture of motorized three-point straining setup and its calibration. a) the complete setup. (**d**) Picture of a flexible substrate with electrodes subjected to strain with the straining setup. Adapted with permission from ref (97). Copyright 2022 WILEY-VCH Verlag GmbH & Co. KGaA, Weinheim. Picture of assembled 4-point setup with a flexible polycarbonate substrate under (**e**) tensile and (**f**) compressive test. Insets show the cartoon illustrations of strained status under tension and compression, respectively. Adapted with permission from ref (98). Copyright 2024, Springer Nature. (**g**) 3D illustration of the experimental setup with gas inlet and outlet (covered by the PDMS) on the pressure chamber and the acrylic holder for fixing the PDMS. (Left-hand side) Illustration of the cross section of the MoS_2_ device, showing that the MoS_2_ is clamped with silicone adhesive and strained on top of the bulging PDMS. (Below) Photograph of the bulging PDMS observed during experiment. Adapted with permission from ref (99). Copyright 2017 American Chemical Society. (**h**) Microscope image of the investigated WS_2_ sample collected with a 50× objective. The magenta square identifies the dome considered for measurements. (Left-hand side) Schematic representation of a 1L-WS_2_ dome. Adapted with permission from ref (48). Copyright 2024 American Chemical Society. (**i**) Suspended MoS_2_ devices and the measurement setup. (a) Scanning electron microscope (SEM) image of typical MoS_2_ devices with suspended channels and contact electrodes. Scale bar is 1 μm. (**j**) Schematic drawing of the setup for direct current electrical characterization of suspended channel MoS_2_ devices under strain. The suspended atomically thin membrane is deformed at the center using an AFM probe attached to a piezo scanner. The vertical displacement of the scanner (z_piezo_) results in the deflection of the cantilever (δ_probe_) and the membrane (δ_mem_). The device is kept under bias voltage V_ds_, whereas the drain current I_ds_ is monitored. Adapted with permission from ref (49). Copyright 2015, American Chemical Society. (**k**) Schematic of the temperature-dependent experiment. Substrate heating causes thermal expansion of the MoS_2_, and due to its larger thermal expansion coefficient, the PDMS substrate induces an additional biaxial strain Adapted with permission from ref (100). Copyright 2015, IOP Publishing. (**l**) Schematic illustration of the experiment setup used for applying in-plane biaxial strain on black phosphorus nanosheet by heating or cooling the polypropylene substrate. Adapted with permission from ref (54). Copyright 2019, Springer Nature Limited.

As mentioned above, commonly for strain engineering, nanosheets are transferred to a flexible polymeric substrate. By utilizing such substrates, the mechanism for nanosheet deformation is reliant on effective strain transfer from polymer to nanosheet.^41^ Through a variety of methodologies, tensile or compressive strains can be uniaxially applied through two-,^42^ three-,^43^ and four-point^5^ bending setups (Figure 3a–d). For homogeneous biaxial straining, nanosheets can be adhered to a polymer cruciform.^44,45^ Alternatively, nanosheets can be suspended across microvoids on a templated rigid substrate in a test chamber. Through the impermeable nature of nanosheets,^46,47^ the chamber’s environmental gas pressure can be used to controllably apply biaxial tensile or compressive strain (Figure 3g). Furthermore, through ion irradiation, monolayer bulges due to an interlayer gas pressure can also be induced on the surface of bulk layered crystals to cause a similar strain event as above (Figure 3h).^48^ Moreover, a precise mode in which suspended nanosheets can be deformed involves nanoindentation with an atomic force microscopy probe (Figure 3i and j).^49^ Additionally, the biaxial effects of environmental pressure on nanosheet band properties can also be investigated on untemplated substrates via pressurized media applying a downward force, such as oil^50^ or a methanol/ethanol mixture.^51^ Alternatively, noncontact methodologies, such as thermal expansion, can also be applied to similarly induce biaxial strain in nanosheets.^52^ Through a mismatch of the thermal expansion coefficient between deposition nanosheets and substrate, biaxial strain that results in tensile or compressive lattice effects can be induced (Figure 3k).^53^ For example, in Figure 3l, black phosphorus (BP) multilayered nanosheets on a polypropylene substrate exposed to heating and cooling cycles induced biaxial straining that was determinable through the thermal expansion coefficient of the substrate.^54^ For all above methodologies, interfacial properties play a critical role in the uniform application of strain.^55^ Due to the potential for nanosheet-substrate interfacial slippage above the vdW limit of ≈1%,^56^ evaporated metal strips are generally required to anchor nanosheets to a substrate.^57^ The advantage of these strips is that they can in turn be applied as electrical contacts. Recent studies^58^ have demonstrated that thermal effects can also play a significant role in modulating the properties of 2D materials, particularly through the activation of photoluminescence via tunable interlayer interactions. For instance, in the SnS/TiS_2_ (SnTiS_3_) superlattice, a naturally occurring van der Waals heterostructure, it was observed that increasing the temperature induces a blue shift in the photoluminescence (PL) peak energy and an increase in PL intensity. This behavior is counter to what is typically observed in 2D semiconductors like MoS_2_ and WS_2_, where temperature generally leads to a redshift and a decrease in PL intensity. This unique response is attributed to the suppression of interlayer charge transfer due to increased lattice mismatch at higher temperatures, highlighting the complex interplay between thermal effects and interlayer interactions in these materials. Understanding these mechanisms is crucial for optimizing the performance of thermally responsive optoelectronic devices based on 2D materials. Table 1 below summarizes the methods used in the literature to apply strain to various 2D materials and how the strain is quantified in each case.

**Table 1:** Strain Application Methods and the Respective Quantification of Strain from Representative Works from the Literature

strain application	material	substrate	strain range	quantification of strain	ref.
AFM nanoindentation	suspended MoS_2_	Si/SiO_2_	Membrane deflection (δ_mem_): 35 nm	Through AFM software, after spring constant calibration	(49)
Bubble formation	MoS_2_	hBN	∼2%	Through ε = h/r (height/radius)	(59)
Lattice mismatch	MoS_2_	WSe_2_	1.59 ± 0.25% (tension)	Through Raman/PL band shifts (calibration according to strain dependent modes of individual monolayers)	(60)
			–1.1 ± 0.18% (compression)		
Bending of flexible substrate	ReSe_2_	Poly(ethylene napthalate)	0.76% (tension)	Through ε = τ/R where 2τ is the thickness of substrate and *R* is the radius of curvature of the substrate (four-point bending)	(61)
			–1.09% (compression)		
Elastic modulus mismatch	MoS_2_	Poly(dimethylsiloxane)	∼2.5%	Through *ε = π*^2^*hδ*(1 – σ^2^)λ^2^ where *h* and σ are the thickness and Passion’s ratio of 2D materials, and δ and λ are the height and width of periodic wrinkles	(62)
Thermal expansion mismatch	Black phosphorus	Polypropylene	biaxial strain: 0.3% (tension-heating)	Through ε = α(*Τ* – *Τ*_0_) where α is the experimentally measured thermal expansion coefficient of the substrate and *T*_0_ is the temperature at zero strain	(54)
			–0.3% (compression-cooling)		
Patterned substrate	MoS_2_	Rippled Si/SiO_2_	∼2.5–3%	Through the observation of characteristic Raman band shifts	(63)
Piezoelectric substrate	MoS_2_	Pb(Mg_1/3_Nb_2/3_)O_3_]_0.7_-[PbTiO_3_]_0.3_	Biaxial strain: –0.04%/100 V	Through ε_∥_ = 0.7ε_⊥_ where ε_⊥_ is the polarization-induced out of plane strain and ε_∥_ is the in-plane strain in the substrate	(64)

### Measuring Strain’s Effect on Nanosheet Properties

Effectively, upon the application of mechanical strain to semiconducting 2D nanosheets, optical and electrical properties can be finely controlled.^65^ Specifically, due to a low density of defects and a lack of dangling bonds, nanosheets are remarkably robust^40,66−68^ in comparison to their bulk semiconducting counterparts.^69,70^ For example, MoS_2_ can withstand strains up to ≈10%, while bulk silicon only ≈1.5%.^56^ Thus, this broader scope for elastic straining in nanosheets lends toward the high level of research interest with regards to their strain engineering. In the simplest of cases, upon the application of strain, bond length will increase for tensile strain and respectively decrease for compressive. Due to a proportionality between bond strength and lattice vibrational frequency, shifts in lattice vibrational modes will occur due to strain effects. Furthermore, tensile and compressive strain will also result in the lattice constant to increase and decrease, respectively. Electronically, valence electron states will alter, manifesting as quantum confinement effects and a widening (compressive) or narrowing (tensile) of optical and electronic bandgap (*E*_g_). Strain’s effect on nanosheet structural and electronic properties can consequently be measured through nondegenerative, high-throughput measurement techniques like Raman spectroscopy and photoluminescence (PL) or differential reflectance spectroscopies to measure lattice and *E*_g_ variations, respectively.^5,71^ Furthermore, through the creation of electronic devices, strain dependent charge transport can be measured via electrical resistance,^49,72^ charge mobilities,^73,74^ or channel voltage.^75,76^

### Quantifying Band Tuning

**Figure 4:**
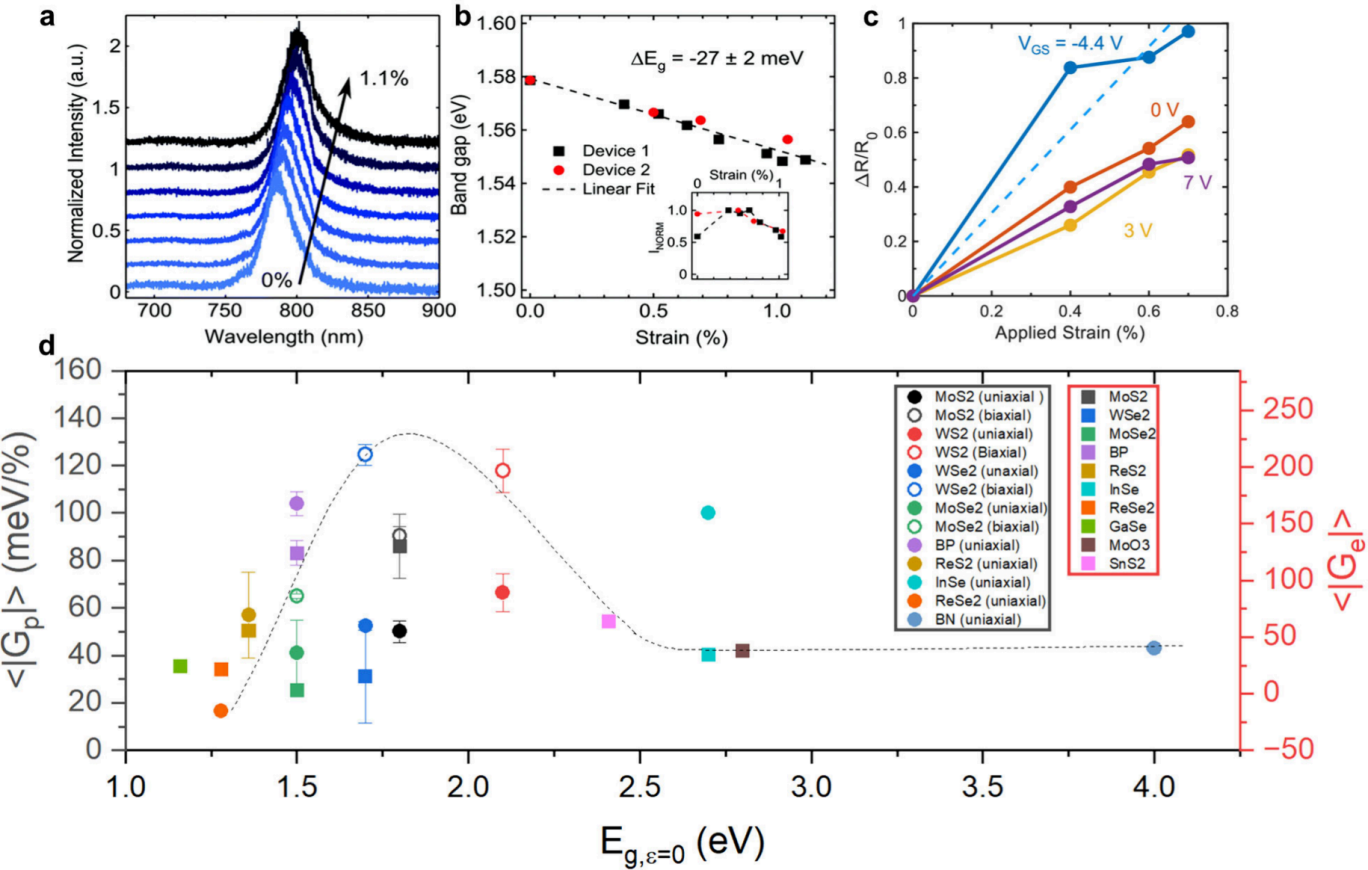
Quantifying nanosheet straining. (**a**) PL spectra for a single-layer MoSe_2_ flake for increasing strains up to 1.1%. (**b**) Change in band gap for two single-layer samples (device 1 is from the spectra in (a)). Adapted with permission from ref (101). Copyright 2016, The Royal Society of Chemistry. (**c**) ΔR/R_0_ vs strain at different gate voltages for the curves in panel for a back-gated monolayer MoS_2_ transistor on a polyethylene naphthalate flexible substrate, with Au source and drain contacts, and an Al_2_O_3_ back-gate dielectric ∼20 nm thick. Adapted with permission from ref (83). Copyright 2022, American Chemical Society. (**d**) Plot of the mean absolute value of photonic (|G_p_|) and piezoresistive (|G_e_|) gauge factors from literature versus zero-strain monolayer bandgap (E_g,ε=0_). Dashed line is a combined Gaussian fit of both gauge factor data sets to reflect literary findings. All data is tabulated in Supporting Information.

With regards to quantifying the tuneability of the band behavior of nanosheets, comparative analysis between material types can be performed through the photonic and piezoresistive gauge factors (*G*_p_ and *G*_e_ respectively). For *G*_p_, the metric is reported as a shift in exciton energies per applied percentage strain and is reported in units of meV/% (Figure 4a and b), while *G*_e_ is described as the fractional change in electrical resistance or current of a nanosheet as a function of absolute strain and is a dimensionless quantity (Figure 4c). Hence, a large value for gauge factor in both instances implies that *E*_g_ rapidly changes with applied strain and *vice versa*. In Figure 4d, when systematically reviewing mean absolute value gauge factor data (<|*G*_p_|> and <|*G*_e_|>) as a function of zero-strain monolayer *E*_g_ (*E*_g,ε=0_), many trends arise. First, it is important to note that substrate plays a vital role with regards to strain transfer to the nanosheet and thus the value of both gauge factors. For instance, MoS_2_ on gold coated polycarbonate^77^ both strains further (i.e., improved strain transfer) and reports a larger value for *G*_p_ than its uncoated counterpart.^78^ Substrate variations naturally leads to quite a large spread in reported data for similar nanosheet types (Figure S3). Specifically, for MoS_2_, |*G*_p_| ranged from ≈29 meV/% to ≈100 meV/%. Moreover, the mode in which strain is applied also greatly effects the rate of change in *E*_g_. In Table S1, biaxially strained nanosheets always reported larger |*G*_p_| values than their uniaxially measured counterparts. For a uniaxially strained WS_2_ monolayer, |*G*_p_| ≈ 59 meV/%.^71^ While for a biaxially strained WS_2_ nanosheet, |*G*_p_| ≈ 135 meV/%.^79^ With regards to |*G*_e_|, to the best of the author’s knowledge, measurements are exclusively measured via uniaxial strain in literature (table S2). Through our master plot, a distinct crossover between the two gauge factors was observed, with the best values for each appearing in the ≈1.7 eV to ≈2.1 eV *E*_g,ε=0_ region. Specifically, values of < |*G*_p_|> for biaxially strained WSe_2_ (≈125 meV/%) and WS_2_ (≈118 meV/%) were found to have the largest band sensitivities. This runs many parallels with experimental^80^ and theoretical^81^ studies which named WSe_2_ and WS_2_ as the most strain sensitive transition metal dichalcogenides as tensile strain in WS_2_ not only reduces the conduction band splitting but also affects the spin relaxation processes,^82^ which are essential for applications in spintronics and valleytronics. The ability to manipulate these dynamics through strain engineering opens new avenues for designing devices that rely on precise control of spin and valley degrees of freedom. For instance, in devices where spin polarization is crucial, applying strain can serve as a mechanism to modulate spin lifetimes and enhance overall device efficiency. Essentially, spin–orbit coupling controlled by optimum atomic composition has a large effect on gauge factor.^80,81^ With regards to < |*G*_e_|>_,_ MoS_2_ reported the largest value for the surveyed materials at ≈130. With a study by Datye et al. reporting the largest overall surveyed |*G*_e_| value of ≈200.^83^ However, the authors note that the *G*_e_ metric appears to be far less reported upon in literature.

### Straintronic Applications

In application, the determination of *G*_p_ and *G*_e_ reflect the unmatched potential straintronics offers with regards to facilitating emerging technologies in telecommunication and health monitoring, respectively. Specifically, using the previously discussed device heterostructures, flexible electronic devices based on nanosheets are enabled. These two figures of merit highlight how the strain modulation of nanosheet band properties can lead to lightweight, conformable, wearable photodetective and bioelectronic devices.^84^ Specifically for semiconducting nanosheets, their strain tunable *E*_g_ offers potential photodetectors with the ability to efficiently absorb light in the visible and near-infrared spectral bandwidth.^85^ For the photoresponsivity of an MoS_2_ monolayer, tensile strain less than 1% resulted in a 3-fold increase in said response.^72^ Additionally, a WS_2_ monolayer directly grown on a porous Si_3_N_4_ substrate via CVD,^86^ resulted in the partial suspension of the nanosheet. A phototransistor based on the as-grown monolayer WS_2_ exhibited a biaxial tensile strain distribution due to the porous nature of the substrate and reported a record high responsivity of 1.58 × 10^5^ A/W and response speed of 40 ms.^86^

Furthermore, this same band tuneability with regards to health monitoring applications facilitates the detection of minute, low strain physiological events, like arterial movements in the skin.^87^ Such measurements would in turn allow for the monitoring of pulse and blood pressure in real time. Generally, for these devices, performance is reliant on strain transfer from a polymeric substrate to a network of semiconducting nanosheets rather than a single nanosheet. However, these devices function on the same principle of applied strain inducing band changes in the nanosheets making up the network.^88^ For PtSe_2_ films grown on polyimide acting as electromechanical piezoresistive sensors, a negative network gauge factor (*G*_net_) up to −85 was observed via tensile straining.^89^ Similarly, for a PdSe_2_ film grown directly on a polyimide substrate presented sensors with *G*_net_ up to −315 ± 2.1 under ∼0.8% strain.^90^ By attaching the PdSe_2_ sensor to the radial artery of the wrist, arterial pulse signals with the typical percussion, tidal, and diastolic waves were observed.

## Conclusion and Outlook

The field of straintronics, particularly through the bandgap engineering of 2D materials, represents a transformative approach to the design and development of advanced electronic devices. This review has underscored the significance of mechanical strain as a tool to finely tune the electronic properties of 2D materials since these have been shown to possess unique structural and electronic characteristics that are highly tunable via strain engineering, making them ideal candidates for a variety of applications in electronics and optoelectronics. A number of studies indicate that even slight alterations in strain can lead to significant changes in material properties, offering a pathway to precisely control device functionalities at a nanoscale level. The ability to adjust the bandgap dynamically through strain not only enhances the performance of existing devices but also paves the way for the development of novel straintronic devices.

Despite the promising advancements in the field, several challenges remain. These include the need for better control over the uniformity of applied strain and the effectiveness of strain transfer from different substrates to the 2D material. Additionally, the majority of research works on straintronics have focused on mechanically exfoliated flakes, produced by a process that is difficult to scale up, even though considerable efforts have been reported recently.^91−93^ Understanding the long-term stability of strained materials under a variety of environmental conditions (e.g., varying temperature and humidity) is another key aspect of straintronics that needs to be evaluated further. With the ongoing advancement of van der Waals and other heterostructures, the integration of strained 2D materials with various components in device architectures is undoubtedly set to become an expanding field. Future research should also focus on scalable and reproducible strain application methods that can be integrated into standard fabrication processes.

In conclusion, as the field of straintronics continues to evolve, it holds the potential to revolutionize the electronic and optoelectronic device landscape. By advancing our understanding of strain effects in 2D materials and overcoming current technological hurdles, we can look forward to the development of more efficient, flexible, and highly tunable electronic systems.

## Supporting Information Available

Figure S1 depicting the total valence charge density of bilayer graphene is plotted along the (110) plane for Bernal stacking from DFT calculations, Figure S2 depicting the densities of states of the carbon atoms for graphene deposited onto different substrates. Figure S3 is a plot of the absolute value of photonic (|G_p_|) and piezoresistive (|G_e_|) gauge factors versus zero-strain monolayer bandgap (E_g,ε_ = 0). Table S1 lists the absolute values for photonic gauge factors for various nanosheet types and Table S2 the piezoresistive gauge factor of various nanosheet types (PDF)
