# Supplementary material for: Bandgap Engineering of 2D Materials toward High-Performing Straintronics
Source: Nano Lett. 2024 Oct 2;24(41):12722–32. doi: 10.1021/acs.nanolett.4c03321 (PMC11487627; doi:10.1021/acs.nanolett.4c03321)
Supplement: Supplementary file 1 — nl4c03321_si_001.pdf [file nl4c03321_si_001.pdf]

Bandgap engineering of 2D materials towards high-performing straintronics

Conor S Boland[1]*, Yiwei Sun[2], Dimitrios G Papageorgiou[2]*

[1]School of Mathematical and Physical Sciences, University of Sussex, Brighton, BN1 9QH, UK

[2]School of Engineering and Materials Science, Queen Mary University, London, E1 4NS, UK

SUPPORTING INFORMATION

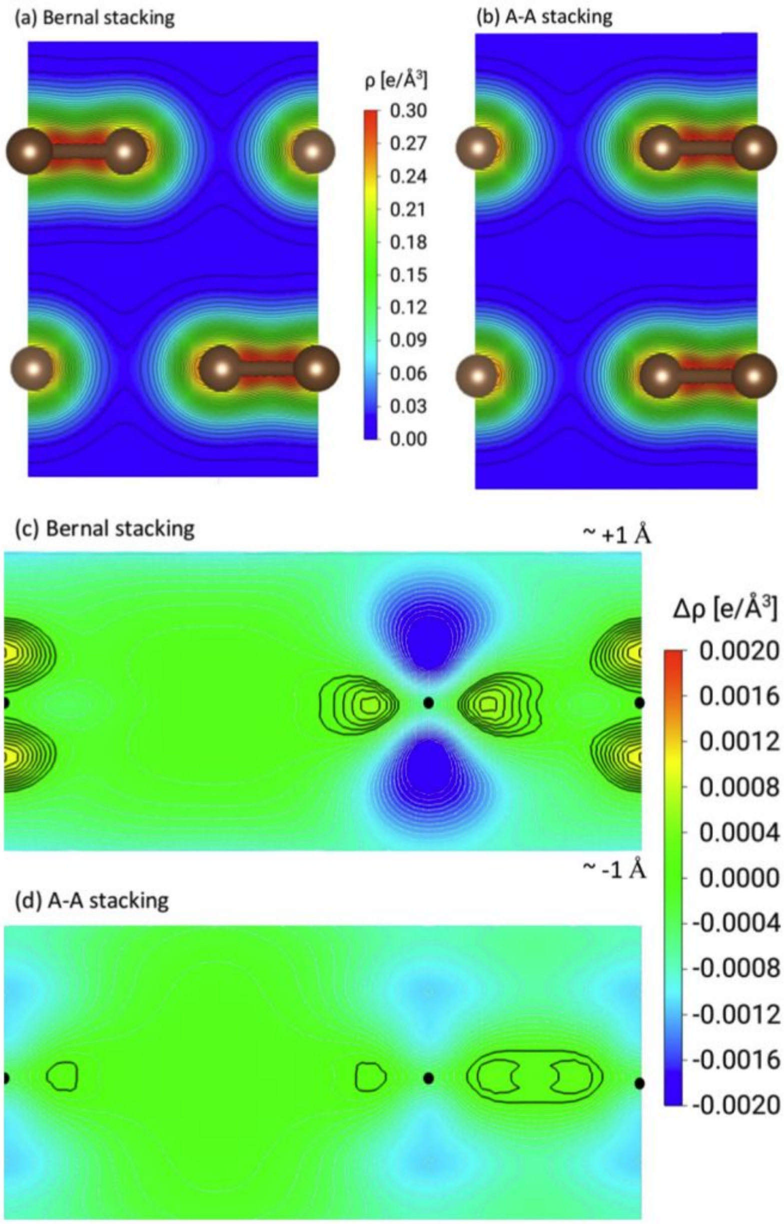

Figure S1: The total valence charge density of bilayer graphene is plotted along the (110) plane for Bernal stacking from DFT calculations. The colour scale is labelled. Positions of atomic cores and *sp*2 bonds are marked. The π and π* states are not spatially separable in the way they

might be in simpler systems.

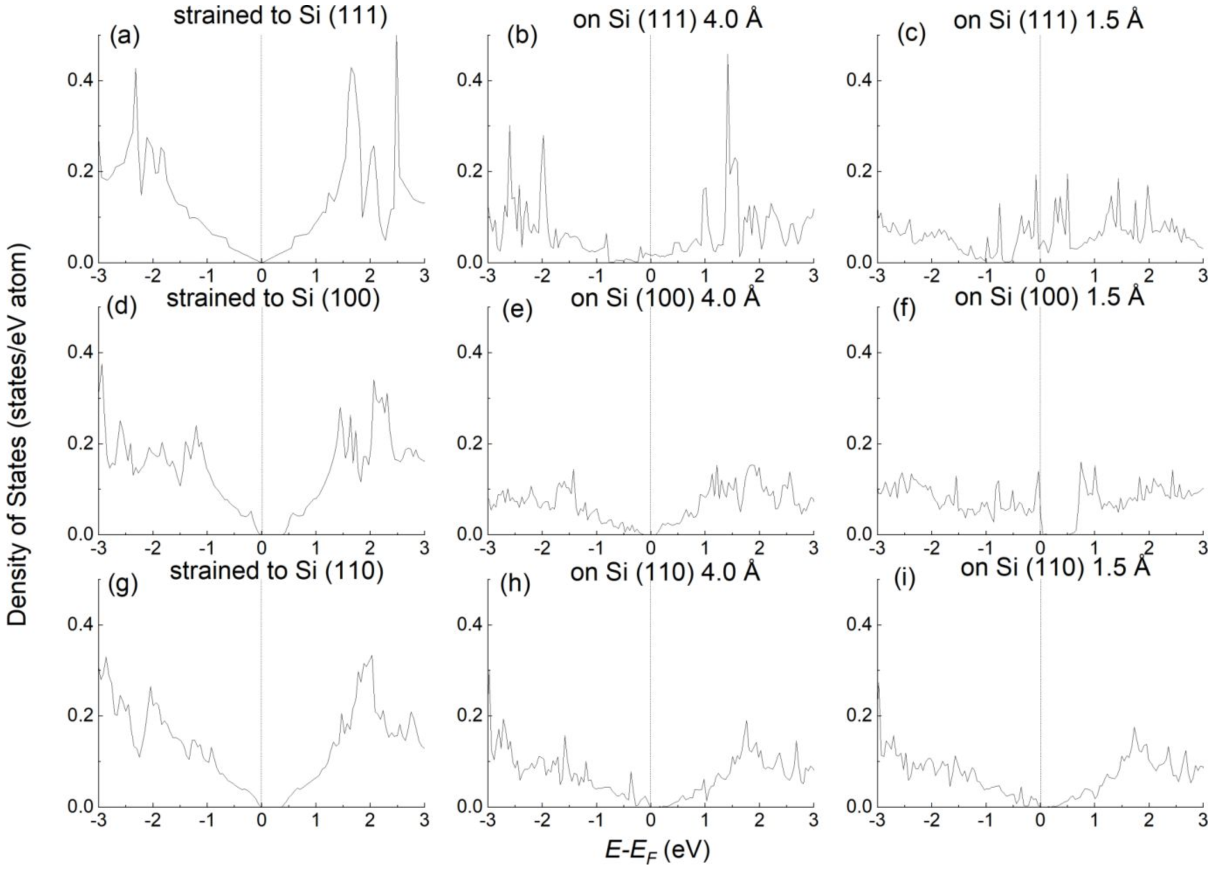

Figure S2: The densities of states (per atom, around the Fermi levels of the systems) of the carbon atoms are plotted, for graphene (a) strained to Si (111) but unsupported, (b) on Si (111) with an initial separation of 4.0 Å, (c) on Si (111) with 1.5 Å, (d) strained to Si (100) but unsupported, (e) on Si (100) with 4.0 Å, (f) Si (100) with 1.5 Å, (g) strained to Si (110) but unsupported, (h) Si (110) with 4.0 Å, and (i) Si (110) with 1.5 Å. Energy is referenced to the Fermi levels, which is shown by the chain-dotted lines at $E - EF = 0$.

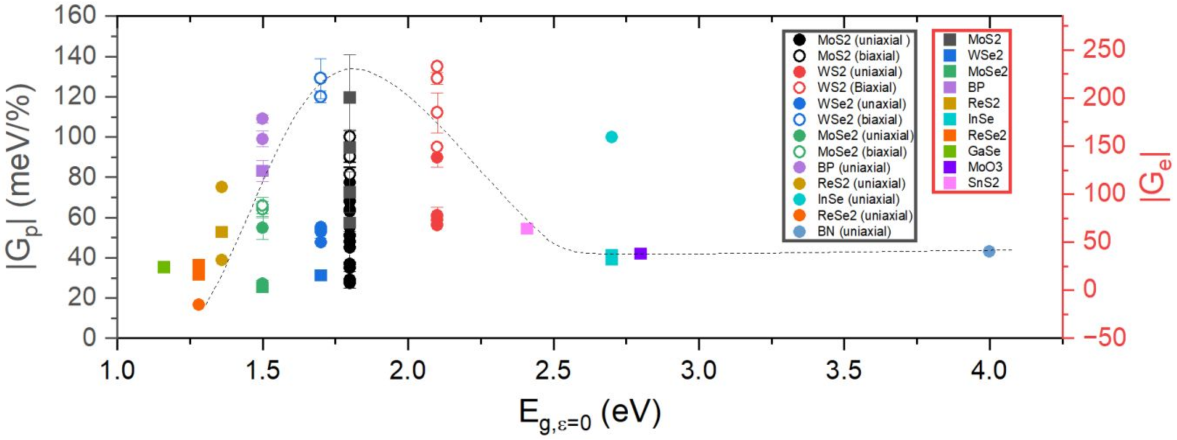

Figure S3. Plot of the absolute value of photonic ($|G_p|$) and piezoresistive ($|G_e|$) gauge factors versus zero-strain monolayer bandgap ($E_{g,\varepsilon=0}$). All data is tabulated in table S1 and S2 respectively.

Reference	Material	Absolute Value of Photonic Gauge Factor (meV/%)	Measurement Type	Notes
1	MoS_2	77.3 ± 10.0	Uniaxial	A exciton
2	MoS_2	51 ± 6	Uniaxial	A exciton
2	MoS_2	29 ± 4	Uniaxial	B exciton
3	MoS_2	63 ± 10	Uniaxial	A exciton
4	MoS_2	37.0 ± 0.4	Uniaxial	A exciton, Reflectance
4	MoS_2	34.8 ± 0.4	Uniaxial	B exciton, Reflectance
4	MoS_2	27.5 ± 0.4	Uniaxial	A exciton, PL
4	MoS_2	55.0 ± 0.4	Uniaxial	B exciton, PL
5	MoS_2	45 ± 7	Uniaxial	A exciton
6	MoS_2	64 ± 5	Uniaxial	A exciton
6	MoS_2	68 ± 5	Uniaxial	B exciton
7	MoS_2	48	Uniaxial	A exciton
8	MoS_2	100 ± 3	Biaxial	Low temp, A exciton
8	MoS_2	90 ± 5	Biaxial	Low temp, B exciton
9	MoS_2	90.2 ± 2.5	Biaxial	A exciton
9	MoS_2	81.5 ± 3.2	Biaxial	B exciton
10	WS_2	61.2 ± 3.8	Uniaxial	A exciton
11	WS_2	58.7 ± 1.4	Uniaxial	A exciton
11	WS_2	89.9 ± 4.9	Uniaxial	Trion exciton
12	WS_2	56	Uniaxial	A exciton
8	WS_2	129 ± 3	Biaxial	Low Temp, A exciton
8	WS_2	112 ± 10	Biaxial	Low Temp, B exciton
12	WS_2	135	Biaxial	A exciton
13	WS_2	95	Biaxial	A exciton
14	WSe_2	47.6	Uniaxial	Calculated, A exciton
10	WSe_2	53.0 ± 3.1	Uniaxial	A exciton

15	WSe_2	54	Uniaxial	A exciton
15	WSe_2	55	Uniaxial	Trion exciton
8	WSe_2	120 ± 3	Biaxial	Low Temp, A exciton
8	WSe_2	129 ± 10	Biaxial	Low Temp, B exciton
10	$MoSe_2$	54.8 ± 5.8	Uniaxial	A exciton
16	$MoSe_2$	27 ± 2	Uniaxial	A exciton
8	$MoSe_2$	64 ± 4	Biaxial	Low Temp, A exciton
8	$MoSe_2$	66 ± 4	Biaxial	Low Temp, B exciton
17	BP	109 ± 2	Uniaxial	Zigzag direction
17	BP	99 ± 4	Uniaxial	Armchair direction
18	ReS_2	75	Uniaxial	Calculated, A exciton
18	ReS_2	39	Uniaxial	Calculated, A exciton
19	InSe	100	Uniaxial	A exciton
20	$ReSe_2$	16.65	Uniaxial	A exciton
21	BN	43	Uniaxial	Simulation

Table S1. Absolute value for photonic gauge factor for various nanosheet types.

Reference	Material	Absolute Value of Piezoresistive Gauge Factor
1	MoS_2	148 ± 19
2	MoS_2	70 ± 3
3	MoS_2	200 ± 45
4	MoS_2	102 ± 21
22	WSe_2	15.2
22	$MoSe_2$	3.1
17	BP	124 ± 11
18	ReS_2	50.14 ± 0.10
18	ReS_2	60.49 ± 0.37
19	InSe	32
19	InSe	36
20	$ReSe_2$	26
20	$ReSe_2$	16.5
22	GaSe	24
23	MoO_3	38 ± 1
22	SnS_2	64

Table S2. Piezoresistive gauge factor of various nanosheet types. All measurements were uniaxial.

References

1. Manzeli, S.; Allain, A.; Ghadimi, A.; Kis, A., Piezoresistivity and Strain-Induced Band Gap Tuning in Atomically Thin Mos2. *Nano Lett.* **2015,** *15* (8), 5330-5335. DOI: 10.1021/acs.nanolett.5b01689.
2. Radatović, B.; Çakıroğlu, O.; Jadriško, V.; Frisenda, R.; Senkić, A.; Vujičić, N.; Kralj, M.; Petrović, M.; Castellanos-Gomez, A., Strain-Enhanced Large-Area Monolayer Mos2 Photodetectors. *ACS applied materials & interfaces* **2024,** *16* (12), 15596-15604. DOI: 10.1021/acsami.4c00458.
3. Datye, I. M.; Daus, A.; Grady, R. W.; Brenner, K.; Vaziri, S.; Pop, E., Strain-Enhanced Mobility of Monolayer Mos2. *Nano Lett.* **2022,** *22* (20), 8052-8059. DOI: 10.1021/acs.nanolett.2c01707.
4. Çakıroğlu, O.; Island, J. O.; Xie, Y.; Frisenda, R.; Castellanos-Gomez, A., An Automated System for Strain Engineering and Straintronics of 2d Materials. *Advanced Materials Technologies* **2023,** *8* (1), 2201091. DOI: 10.1002/admt.202201091.
5. Conley, H. J.; Wang, B.; Ziegler, J. I.; Haglund, R. F., Jr.; Pantelides, S. T.; Bolotin, K. I., Bandgap Engineering of Strained Monolayer and Bilayer Mos2. *Nano Lett.* **2013,** *13* (8), 3626-3630. DOI: 10.1021/nl4014748.
6. He, K.; Poole, C.; Mak, K. F.; Shan, J., Experimental Demonstration of Continuous Electronic Structure Tuning Via Strain in Atomically Thin Mos2. *Nano Lett.* **2013,** *13* (6), 2931-2936. DOI: 10.1021/nl4013166.
7. Zhu, C. R.; Wang, G.; Liu, B. L.; Marie, X.; Qiao, X. F.; Zhang, X.; Wu, X. X.; Fan, H.; Tan, P. H.; Amand, T.; Urbaszek, B., Strain Tuning of Optical Emission Energy and Polarization in Monolayer and Bilayer Mos${}_{2}$. *Physical Review B* **2013,** *88* (12), 121301. DOI: 10.1103/PhysRevB.88.121301.
8. Henríquez-Guerra, E.; Li, H.; Pasqués-Gramage, P.; Gosálbez-Martínez, D.; D'Agosta, R.; Castellanos-Gomez, A.; Calvo, M. R., Large Biaxial Compressive Strain Tuning of Neutral and Charged Excitons in Single-Layer Transition Metal Dichalcogenides. *ACS applied materials & interfaces* **2023,** *15* (49), 57369-57378. DOI: 10.1021/acsami.3c13281.
9. Carrascoso, F.; Frisenda, R.; Castellanos-Gomez, A., Biaxial Versus Uniaxial Strain Tuning of Single-Layer Mos2. *Nano Materials Science* **2022,** *4* (1), 44-51. DOI: 10.1016/j.nanoms.2021.03.001.
10. Mennel, L.; Paur, M.; Mueller, T., Second Harmonic Generation in Strained Transition Metal Dichalcogenide Monolayers: Mos2, Mose2, Ws2, and Wse2. *APL Photonics* **2018,** *4* (3), 034404. DOI: 10.1063/1.5051965.
11. Wang, F.; Li, S.; Bissett, M. A.; Kinloch, I. A.; Li, Z.; Young, R. J., Strain Engineering in Monolayer Ws2 and Ws2 Nanocomposites. *2d Mater* **2020,** *7* (4), 045022. DOI: 10.1088/2053-1583/ababf1.
12. Oliva, R.; Wozniak, T.; Faria, P. E., Jr.; Dybala, F.; Kopaczek, J.; Fabian, J.; Scharoch, P.; Kudrawiec, R., Strong Substrate Strain Effects in Multilayered Ws2 Revealed by High-Pressure Optical Measurements. *ACS applied materials & interfaces* **2022,** *14* (17), 19857-19868. DOI: 10.1021/acsami.2c01726.
13. Frisenda, R.; Drüppel, M.; Schmidt, R.; Michaelis de Vasconcellos, S.; Perez de Lara, D.; Bratschitsch, R.; Rohlfing, M.; Castellanos-Gomez, A., Biaxial Strain Tuning of the Optical Properties of Single-Layer Transition Metal Dichalcogenides. *npj 2D Materials and Applications* **2017,** *1* (1), 1-7. DOI: 10.1038/s41699-017-0013-7.
14. Aslan, B.; Deng, M.; Heinz, T. F., Strain Tuning of Excitons in Monolayer ${\Mathrm{Wse}}_{2}$. *Physical Review B* **2018,** *98* (11), 115308. DOI: 10.1103/PhysRevB.98.115308.

15. Schmidt, R.; Niehues, I.; Schneider, R.; Drüppel, M.; Deilmann, T.; Rohlfing, M.; Vasconcellos, S. M. d.; Castellanos-Gomez, A.; Bratschitsch, R., Reversible Uniaxial Strain Tuning in Atomically Thin Wse2. *2d Mater* **2016,** *3* (2), 021011. DOI: 10.1088/2053-1583/3/2/021011.
16. O. Island, J.; Kuc, A.; H. Diependaal, E.; Bratschitsch, R.; Zant, H. S. J. v. d.; Heine, T.; Castellanos-Gomez, A., Precise and Reversible Band Gap Tuning in Single-Layer Mose 2 by Uniaxial Strain. *Nanoscale* **2016,** *8* (5), 2589-2593. DOI: 10.1039/C5NR08219F.
17. Zhang, Z.; Li, L.; Horng, J.; Wang, N. Z.; Yang, F.; Yu, Y.; Zhang, Y.; Chen, G.; Watanabe, K.; Taniguchi, T.; Chen, X. H.; Wang, F.; Zhang, Y., Strain-Modulated Bandgap and Piezo-Resistive Effect in Black Phosphorus Field-Effect Transistors. *Nano Lett.* **2017,** *17* (10), 6097-6103. DOI: 10.1021/acs.nanolett.7b02624.
18. An, C.; Xu, Z.; Shen, W.; Zhang, R.; Sun, Z.; Tang, S.; Xiao, Y.-F.; Zhang, D.; Sun, D.; Hu, X.; Hu, C.; Yang, L.; Liu, J., The Opposite Anisotropic Piezoresistive Effect of Res2. *ACS Nano* **2019,** *13* (3), 3310-3319. DOI: 10.1021/acsnano.8b09161.
19. Chen, L.; Liang, D.; Yu, Z.; Li, S.; Feng, X.; Li, B.; Li, Y.; Zhang, Y.-W.; Ang, K.-W. In *Ultrasensitive Flexible Strain Sensor Based on Two-Dimensional Inse for Human Motion Surveillance*, 2019 IEEE International Electron Devices Meeting (IEDM), 2019/12//; 2019; pp 26.26.21-26.26.24.
20. Liu, Y.; Li, X.; Guo, Y.; Yang, T.; Chen, K.; Lin, C.; Wei, J.; Liu, Q.; Lu, Y.; Dong, L.; Shan, C., Modulation on the Electronic Properties and Band Gap of Layered Rese2 Via Strain Engineering. *Journal of Alloys and Compounds* **2020,** *827*, 154364. DOI: 10.1016/j.jallcom.2020.154364.
21. Lechifflart, P.; Paleari, F.; Attaccalite, C., Excitons under Strain: Light Absorption and Emission in Strained Hexagonal Boron Nitride. *SciPost Physics* **2022,** *12* (5), 145. DOI: 10.21468/SciPostPhys.12.5.145.
22. Yan, W.; Fuh, H.-R.; Lv, Y.; Chen, K.-Q.; Tsai, T.-Y.; Wu, Y.-R.; Shieh, T.-H.; Hung, K.-M.; Li, J.; Zhang, D.; Ó Coileáin, C.; Arora, S. K.; Wang, Z.; Jiang, Z.; Chang, C.-R.; Wu, H.-C., Giant Gauge Factor of Van Der Waals Material Based Strain Sensors. *Nature Communications* **2021,** *12* (1), 2018. DOI: 10.1038/s41467-021-22316-8.
23. Puebla, S.; Li, H.; Çakıroğlu, O.; Sánchez-Viso, E.; Munuera, C.; D’Agosta, R.; Castellanos-Gomez, A., Strain Tuning Moo3 Vibrational and Electronic Properties. *npj 2D Materials and Applications* **2024,** *8* (1), 1-9. DOI: 10.1038/s41699-024-00442-3.
